# Resequencing Microarray Technology for Genotyping Human Papillomavirus in Cervical Smears

**DOI:** 10.1371/journal.pone.0109301

**Published:** 2014-11-10

**Authors:** Nicolas Berthet, Michael Falguières, Claudia Filippone, Chloé Bertolus, Christine Bole-Feysot, Sylvain Brisse, Antoine Gessain, Isabelle Heard, Michel Favre

**Affiliations:** 1 Institut Pasteur, Epidémiologie et Physiopathologie des Virus Oncogènes, 75724 Paris Cedex 15, France; 2 Centre National de la Recherche Scientifique, UMR 3569, 75724 Paris Cedex 15, France; 3 Institut Pasteur, Centre National de Référence des Papillomavirus, 75724 Paris Cedex 15, France; 4 AP-HP, Hôpital Pitié-Salpêtrière, Service de Chirurgie Maxillo-Faciale et Stomatologie, 75013 Paris, France; 5 UPMC, Université Paris 06, CIMI-Paris, UMRS CR7, INSERM U1135, 75005, Paris, France; 6 Imagine, Institut des maladies génétiques - Plateforme Génomique, Hôpital Necker - Enfants Malades, 75743 Paris cedex 15, France; 7 Institut Pasteur, Plate-forme Génotypage des Pathogènes et Santé Publique, 75724 Paris Cedex 15, France; 8 UPMC Université Paris 06, Groupe hospitalier Pitié-Salpêtrière, Paris Cedex 13, France; 9 Institut Pasteur, Unité de Génétique, Papillomavirus et Cancer humain, 75724 Paris Cedex 15, France; University of Houston, United States of America

## Abstract

There are more than 40 human papillomaviruses (HPVs) belonging to the alpha genus that cause sexually transmitted infections; these infections are among the most frequent and can lead to condylomas and anogenital intra-epithelial neoplasia. At least 18 of these viruses are causative agents of anogenital carcinomas. We evaluated the performance of a resequencing microarray for the detection and genotyping of alpha HPV of clinical significance using cloned HPV DNA. To reduce the number of HPV genotypes tiled on microarray, we used reconstructed ancestral sequences (RASs) as they are more closely related to the various genotypes than the current genotypes are among themselves. The performance of this approach was tested by genotyping with a set of 40 cervical smears already genotyped using the commercial PapilloCheck kit. The results of the two tests were concordant for 70% (28/40) of the samples and compatible for 30% (12/40). Our findings indicate that RASs were able to detect and identify one or several HPV in clinical samples. Associating RASs with homonym sequences improved the genotyping of HPV present in cases of multiple infection. In conclusion, we demonstrate the diagnostic potential of resequencing technology for genotyping of HPV, and illustrate its value both for epidemiological studies and for monitoring the distribution of HPV in the post-vaccination era.

## Introduction

More than 170 human papillomavirus (HPVs) have been characterized and they are classified into the alpha, beta, gamma, mu, and nu genera [1]. Some alpha papillomaviruses presenting a mucous tropism cause some of the most frequent sexually transmitted infections: around 300 million women worldwide carry a HPV infection of the uterine cervix. About 40 HPV genotypes able to infect genital mucous membranes have been identified. Low-risk mucosal HPVs (LR HPVs) are responsible for condylomas found in 1–2% of the sexually active population. High-risk HPVs (HR HPVs) are causative agents of more than 80% of high-grade cervical intra-epithelial neoplasia [2]. Persistent lesions associated with HR HPVs may evolve towards invasive cervical carcinomas. HPV DNA sequences are detected in the vast majority of adenocarcinomas, adenosquamous carcinomas and squamous cell carcinomas of the cervix, all of which are preceded by premalignant lesions [3]. HR HPVs also contributes to the pathogenesis of other anogenital and aero-digestive tract cancers [4].

The need for effective screening for cervical cancer is now recognized worldwide and testing for HPVs is a potentially powerful approach to the detection of cervical or anogenital lesions. Other than the hybrid capture II and the Cervista detection tests [5], most of the available tests for HPVs are based on PCR methods using degenerate or consensus primers. All these methods allow the detection, and some allow genotyping, of a wide spectrum of HPVs. In addition to their clinical value, robust methods of detection are required for epidemiological studies of HPV infections. In particular, they could be used to study the prevalence and distribution of circulating HPV genotypes in vaccinated and non-vaccinated populations.

Several low or high-density microarrays have been developed and successfully used for the detection of several viral families such as *Papillomaviridae*. Most of them are based on long oligonucleotide probes and virus identification is based only on hybridization patterns. This approach leads to a lack of information for pathogen identification at the single-pair resolution level [6], [7]. However, this limitation has since been overcome with the use of the resequencing microarrays (RMAs) method. Resequencing microarray (RMA) technology involves generating a DNA/RNA sequence corresponding to the pathogen(s) present in a sample in a single assay. It allows detection of a sequence with a tolerance of divergence of up to 10–15% from those tiled on the microarray [8], [9], [10]. We have previously used RMAs for the detection of a large panel of viral and bacterial pathogens by resequencing the hybridised DNA. This approach has been successfully applied to diverse clinical situations such as the establishment of a rapid laboratory diagnostic test for pandemic influenza viruses [11], [12], the characterization of different strains of the monkeypox virus [13], [14], the genotyping of several members of the Rhabdoviridae family [8] and the identification and characterization of arboviruses and hemorrhagic fever viruses in biological samples [15], [16].

The aim of this study was to develop a multiplex bioassay method, based on RMA technology, for the detection of mucosal HPV genotypes of public health importance. Because of the wide genetic diversity of HPVs and to reduce the number of genotypes tiled on the microarray, we inferred reconstructed ancestral sequences (RASs); these sequences are more closely related to different genotypes than such genotypes are to each other. The advantages of using reconstructed ancestral sequences for RMAs has been described and illustrated using pathogen identification with *Enterobacteriaceae rpoB* sequences used as models [17]. We went on to apply the RMA method developed to detection of HPV DNA sequences in clinical samples.

## Materials and Methods

### Principle and content of resequencing microarray

A third generation resequencing microarray, named VirID v3.0, containing 724 viral sequences distributed into 27 families and 85 genera (Table S1), was used in this study. Among the many viral sequences tiled, a set of 38 different HPV sequences was used. The size of the tiled region ranged from 455 to 482 nucleotides corresponding to part of the L1 open reading frame. This set includes sequences in the following genotypes: HPV6, 11, 16, 18, 26, 30, 31, 33, 34, 35, 40, 42, 43, 44, 45, 51, 53, 54, 56, 58, 59, 65, 66, 67, 68, 69, 70, 73, 74, 82, 85, 88, 91, 95, 97, 101, 103 and 108. In addition, ten reconstructed ancestral sequences (RASs) were tiled on the microarray. These sequences are described in Tables S1 and S2. For each sequence tiled, the principle of RMA is to interrogate each single base of the unknown sequence to be detected with a set of eight appropriate 25-mer probes. Two probes among the eight (four for each sense of the selected sequence) correspond to perfect matches at the central (13th) position of the probe, and the other six probes represent all possible mismatches at the same position.

### Ancestral sequences reconstruction

RASs were designed based on phylogenetic analysis of nucleotide sequences of a portion of 416 bases in the *L1* ORF of 38 alpha-*HPV*; there were neither insertions nor deletions in this stretch. A neighbor-joining tree was obtained using software BioNumerics v5.10 (Applied-Maths, Belgium). Ancestral sequences were reconstructed by maximum likelihood using the PAML v4 software [18]. The nucleotide substitution model used was K80 with gamma (number of categories of distinct substitution rates) and kappa (transition/transversion ratio) parameters estimated.

### Extraction and amplification of HPV DNA

Viral DNA was obtained from cloned HPV DNA and from cervical smears provided by the National Reference Laboratory for Human Papillomavirus at Institut Pasteur (Paris). The cloned HPV DNA used for validation assays was amplified and purified as previously described [19]. Total DNA was extracted from cervical smears using the NucleoSpin Tissue extraction kit according to the recommendations of the supplier (Macherey Nagel, Hoerdt, France). Following extraction, samples were digested with Plasmid-Safe DNase for 12 hours at 37°C according to the manufacturer’s instructions to enrich for circular viral DNA (Epicentre). All DNAs were amplified using the Repli-g Mini Kit according to the manufacturer’s instructions (Qiagen). The concentration of amplified DNAs was determined with the Quant-it kit (Life technologies) and aliquots of 10 ug were used for hybridization in RMA assays.

### Ethical considerations

Liquid-based cytology (LBC) samples were collected from women attending organized cervical screening in 16 pilot sites in France. After completion of cytology, residual LBC samples were anonymized and sent to the French HPV Reference Laboratory for genotyping. The residual material would otherwise have been discarded. According to French regulations of biomedical research, a written or verbal informed consent and an ethical approval are not required for such studies. However, the study was given favorable ethical opinion by the Comité de Recherche Clinique at the Institut Pasteur (Avis n° 200-51). The collection of human samples was declared to the French Research Ministry (n°DC-2010-1197, collection n°1) in accordance with French regulations.

### Hybridization on RMA and data analysis

Amplified DNA was fragmented and labeled using the GeneChip Resequencing Assay Kit (Affymetrix Inc., Santa Clara, CA). After overnight hybridization at 45°C, the RMA was washed, stained and scanned according to Affymetrix’ instructions. The raw image file (.DAT), obtained after the scan, was converted to a fluorescence intensity file (.CEL). Bases were called by the GeneChip Sequence Analysis Software (GSEQ v 4.1) which uses a derivative of the ABACUS base-calling algorithm [20]. This algorithm consists of an automated statistical method that analyses raw RMA hybridization data and optimizes the base-calling process. For each base-call, a quality score is calculated from the difference between the best fitting model and the second best one. If this score is below a chosen threshold, an undetermined base “N” is assigned to that position. Sequences were output in FASTA format; for each HPV sequence obtained, the call rate value was calculated as the ratio between the number of bases determined (A or T or C or G) and the entire sequence length. The accuracy of the RMA process was determined as the ratio between the number of correctly determined bases and the total number of determined bases, by comparison with the known sequence of the strains tested.

### Real-time PCR for HPV16 and HPV18

Quantitative PCR tests for HPV16 and HPV18 were performed using DNA obtained from cervical smears (5 ul) or amplified products (1/1000 dilution). The DNA was mixed with 100 pmol of each primer (HPV16-for 5′ GAACCGAAACCGGTTAGTATAA 3′; HPV16-rev 5′ ATGTATAGTTGTTTGCAGCTCTGT 3′; HPV18-for 5′ GGACCGAAAACGGTGTATATAA 3′; HPV18-rev 5′ CAGTGAAGTGTTCAGTTCGGT 3′) and 15 pmol of a probe (HPV16-probe 5′ Fam-CATTTTATGCACCAAAAGAGAACTGCAATGTTTC-BHQ13′; HPV18-probe 5′ Tamra ATGTGAGAAACACACCACAATACTATGGCGCG BHQ2 3′) in a final reaction volume of 20 µl, using a LC Taqman Master kit (Roche Diagnostic, Inc.). The LightCycler qPCR assay was performed as described by Schmitz et al. [21].

### PapilloCheck HPV genotyping test

Clinical samples were tested using the PapilloCheck HPV genotyping kit (Greiner BioOne, Frickenhausen, Germany). This test involves PCR amplification with fluorescent primers (CY5 fluorophore) specific for the HPV E1 gene and the cellular housekeeping gene ADAT1. The amplification products are then hybridized to a DNA chip containing sequences for 18 high-risk genotypes (HPV 16, 18, 31, 33, 35, 39, 45, 51, 52, 53, 56, 58, 59, 66, 68, 70, 73, 82) and six low-risk genotypes (HPV 6, 11, 40, 42, 43 and 54/55). The PapilloCheck test was used according to the recommendations of the supplier.

## Results

### 1. Validation of RMA using cloned HPV DNA

**Figure 1:**
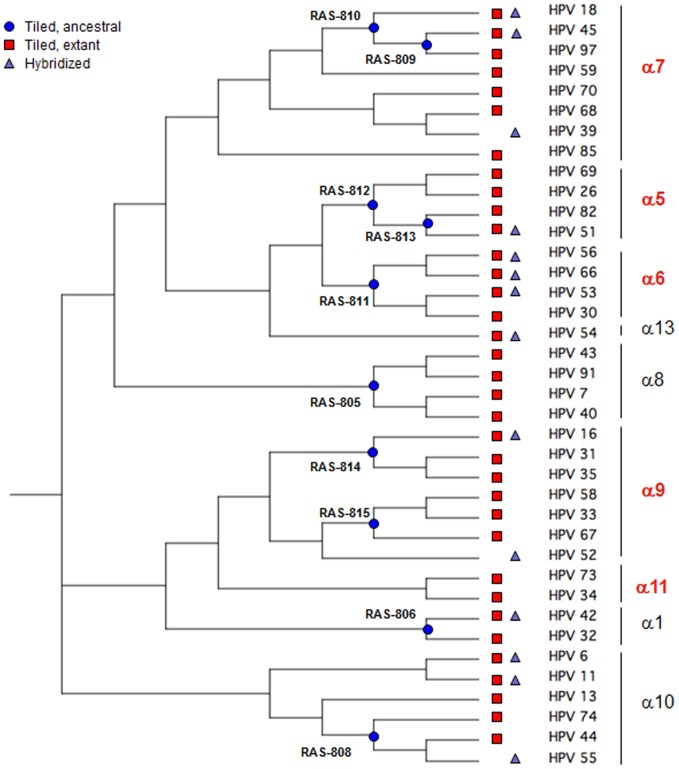
Phylogenetic tree of 38 alpha HPV isolates, constructed to assess the resequencing microarray approach. The tree was constructed from the 416–base sequences of part of the L1 ORF in the different HPVs. The tree was generated by the neighbor-joining method using BioNumerics v5.10 software (Applied-Maths, Belgium). HPV sequences tiled on RMA (red squares), location of RASs on the tree (blue circles) and cloned HPVs tested with RMAs (green triangles) are indicated.

The third generation of the “VirID” RMA (v3.0) was used for the detection and identification of a broad panel of HPV sequences. Thirty-eight HPV sequences were selected and tiled on the microarray, including 32 from the α genus (HPV6, 11, 16, 18, 26, 30, 31, 33, 34, 35, 40, 42, 43, 44, 45, 51, 53, 54, 56, 58, 59, 66, 67, 68, 69, 70, 73, 74, 82, 85, 91, and 97) and covering all the nine families found in cervical lesions and condylomas. The other six sequences belonged to the γ genus (HPV65, 88, 95, 101, 103, and 108). Reconstructed Ancestral Sequences (RAS) were designed through phylogenetic analysis of selected α *genus* sequences. Sequences at nodes at different phylogenetic depths on the tree were chosen as RASs (Fig. 1); we checked that all the RASs selected branched directly or very closely to their respective node, with near-zero branch lengths.

Of the 38 HPV sequences deposited on the chip, 11 α-HPV sequences and one γ-HPV sequence were tested using cloned HPV genomes (Table 1): all the 12 sequences were detected with call rate values between 92.1 and 97.7%, with a resequencing accuracy of 98.6 to 100%. Confirmation of the HPV genotype was obtained following BLASTN analysis, with scores between 704 (for HPV54) to 809 (for HPV108) (Table 1).

**Table 1:** Resequencing microarray assays with cloned HPV DNA.

HPV species	HPV genotype	Call rate^a^ (%)	Accuracy^b^ (%)	BLAST score^c^
α1	42	96.5	99.5	780
α5	51	94.4	99.3	749
α6	53	93.6	98.6	717
	56	95.7	99.5	751
	66	95.4	100	751
α7	18	94.5	99.5	765
	45	94.2	99.8	764
α9	16	94.9	100	767
α10	6	94.7	100	758
	11	94.4	100	738
α13	54	92.1	100	704
γ	108	97.7	100	809

a Ratio between the number of bases determined and the sequence length.

b Ratio between the number of correctly determined bases and the total number of determined bases.

c The score of the highest scoring high-scoring segment pair (HSP) from that database sequence.

### 2. Genotyping of HPVs with homonymous sequences

Because several sequences were tiled on the chip, we tested whether each HPV genotype could be detected following hybridization either to its homonymous sequence, or to related sequences belonging to the same or different species. Various cloned HPVs were tested, alone or in mixtures with HPVs belonging to the same or different species (Tables 2 and 3). HPV16 and HPV66 were perfectly identified by their homonymous sequences, whether they were on their own or in mixtures; they were both identified by non-homonymous sequences, including HPV30 and HPV33. HPV66 was also detected by the sequences of HPV53, HPV56 and HPV82, whereas HPV16 was detected by the sequences of HPV45 and HPV59 (Table 2). With a mixture of both HPV16 and HPV66, only the most closely related HPV was detected by a non-homonymous sequence (Table 2). HPV18 was tested alone and in mixtures with HPV45 or HPV53: the three viruses were perfectly detected by their corresponding homonymous sequences. Moreover, they were simultaneously identified by several other non-homonymous sequences (Table 3): for example, when two HPVs belonging to the same species (such as HPV18 and HPV45) were tested together, no competition was observed for their identification either by homonymous or related sequences. Mixtures containing HPV16, 51, 66, and 108 (Table 2) or HPV6, 18, 42, and 53 (Table 3) were similarly studied, and in all cases the various HPVs were distinguished and identified by both homonymous and non- homonymous sequences.

**Table 2:** Identification of HPV16 and other HPVs with homonymous and non homonymous sequences.

		Cloned HPV tested											
		16			66			16+66			16+66+51+108		
Species	TiledHPV	Call ratevalue^a^	NBlastscore	HPVidentified	Call ratevalue^a^	NBlastscore	HPVidentified	Call ratevalue^a^	NBlastscore	HPVidentified	Call ratevalue^a^	NBlastscore	HPVidentified
α5	51	15.2	Neg	N.A.	12.8	Neg	N.A.	22.5	Neg	N.A.	**94.4**	**749**	**51**
	82	18.7	Neg	N.A.	13.0	53.6	66	21.7	Neg	N.A.	45.3	147	51
α6	56	24.1	Neg	N.A.	41.4	134	66	46.6	75.2	66	46.0	118	66
	53	17.1	Neg	N.A.	31.9	59	66	34.7	Neg	N.A.	38.5	69.8	66
	**66**	28.4	Neg	N.A.	**95.4**	**751**	**66**	**91.3**	**677**	**66**	**95.4**	**751**	**66**
	30	26.9	51.8	16	29.9	101	66	41.9	145	66	36.9	205	66
α7	45	21.6	53.6	16	12.9	Neg	N.A.	25.3	59	Und.	22.4	60.8	Und.
	59	23.9	44.6	16	10.3	Neg	N.A.	25.6	Neg	N.A.	26.4	41	16
α9	**16**	**94.9**	**767**	**16**	23.1	77	66	**90.3**	**688**	**16**	**94.9**	**767**	**16**
	33	25.2	50	16	20.7	44.6	66	32.4	44.6	16	32.6	94.5	16
	35	29.6	Neg	N.A.	14.8	59	66	33.8	101	16	30.8	64.4	16
α10	74	22.8	46.4	16	9.8	Neg	N.A.	22.9	44.6	16	22.6	48.2	16
α13	54	23.3	44.6	16	12.7	Neg	N.A.	24.4	46.4	Und.	24.6	50	Und.
γ6	108	18.1	Neg	N.A.	14.1	Neg	N.A.	18.9	Neg	N.A.	**97.7**	**809**	**108**

a Ratio between the number of bases determined and the sequence length.

Values in bold correspond to the call rate value, NBlast score and result of BLAST of HPVs tested with the homonymous sequence. Neg: Negative; N.A: Not applicable; Und.: Undetermined.

**Table 3:** Identification of HPV18 and other HPVs with homonymous and non homonymous sequences.

		Cloned HPV											
		18			18+45			18+53			18+53+6+42		
Species	TiledHPV	Call ratevalue^a^	NBlastscore	HPVidentified	Call ratevalue^a^	NBlastscore	HPVidentified	Call ratevalue^a^	NBlastscore	HPVidentified	Call ratevalue^a^	NBlastscore	HPVidentified
α1	42	11.2	Neg	N.A.	12.5	Neg	N.A.	23.5	Neg	N.A.	**96.6**	**780**	**42**
α6	53	4	Neg	N.A.	9.5	Neg	N.A.	**92.7**	**702**	**53**	**93.6**	**717**	**53**
	56	10.6	Neg	N.A.	18.0	Neg	N.A.	28.0	46.4	53	29.5	41	53
α7	18	**96.6**	**801**	**18**	**93.7**	**751**	**18**	**89.1**	**666**	**18**	**94.5**	**765**	**18**
	45	47.9	168	18	**94.2**	**764**	**45**	31.3	136	18	52.9	204	18
	59	20.2	Neg	N.A.	27.5	46.4	Und.	17.0	Neg	N.A.	26.9	Neg	N.A.
	68	20.8	75.2	18	28.3	123	18	22.7	57.2	18	27.8	128	18
	70	21.4	Neg	N.A.	31.9	62.8	45	23.1	51.8	53	31.5	Neg	N.A.
	97	41.0	112	18	63.0	262	45	29.4	Neg	N.A.	51.9	211	18
α8	40	17.2	Neg	N.A.	26.0	Neg	N.A.	14.3	Neg	N.A.	20.0	Neg	N.A.
	43	11.5	Neg	N.A.	27.7	55.4	Und.	13.2	Neg	N.A.	23.0	42.8	Und.
α9	16	17.1	46.4	18	21.8	51.8	18	16.9	Neg	N.A.	32.6	77	6
	31	15.0	69.8	18	23.3	64.4	Und	19.9	57.2	53	28.8	122	Und.
	33	14.5	Neg	N.A.	21.7	50	18	19.4	42.8	53	26.4	66.2	6
	35	16.5	Neg	N.A.	25.3	46.4	18	17.3	Neg	N.A.	38.3	48.2	42
	58	10.6	Neg	N.A.	16.6	Neg	N.A.	13.2	Neg	N.A.	20.2	Neg	N.A.
	67	23.0	62.6	18	26.4	112	18	18.9	Neg	N.A.	32.1	98.2	18
α10	6	17.4	54.8	18	21.3	60.8	Und.	23.0	Neg	N.A.	**94.7**	**758**	**6**
α11	73	15.9	Neg	N.A.	19.2	57.2	18	13.4	Neg	N.A.	29.2	42.8	18
α13	54	21.1	Neg	N.A.	28.2	103	45	16.8	Neg	N.A.	34.1	Neg	N.A.

a Ratio between the number of bases determined and the sequence length.

Values in bold correspond to the call rate value, NBlast score and result of BLAST of HPVs tested with the homonymous sequence. Neg: Negative; N.A: Not applicable; Und.: Undetermined.

### 3. HPV genotyping with reconstructed ancestral sequences

We wanted to determine whether all HPVs belonging to a single species could be detected with a RAS specific for this species. Within any family, the RAS is more closely related to the sequences of the various HPVs than the sequences of the HPVs are to each other (data not shown). We tested HPV53, 56 and 66, all members of species alpha 6, and call rates of 59.1 to 75.7 were obtained with RAS-811 (Table 4). These values were much higher than those observed with non-homonymous sequences: 29.5 to 46.0. BLAST analyses of all the sequences obtained confirmed the identification of the genotypes in all cases.

**Table 4:** Identification of HPVs belonging to the α6 family based on homonymous and reconstructed ancestral sequences.

		HPV tested		
HPV sequence tiled on RMA		53	56	66
**HPV53**	Call rate^a^	**93.6** a	35.6	38.5
	BLAST score	717	111	69.8
**HPV56**	Call rate^a^	29.5	**95.7**	46
	BLAST score	41	751	118
**HPV66**	Call rate^a^	34.9	43.9	**95.4**
	BLAST score	42.8	145	751
**RAS_811**	Call rate^a^	59.1	71.2	75.7
	BLAST score	107	197	306

a The call rate value for HPVs tested with its homonymous sequence is indicated in boldface.

We next investigated whether HPVs that belong to different species could be identified with RASs. We tested a mixture of HPV16, 51, 66 and 108 (mixture A) and one of HPV6, 18, 42 and 53 (mixture B) with reconstructed RASs tiled on the microarray (Table 5). HPV16 belonging to the alpha 9 species was detected by RASs of the same alpha 9 species (RAS 814 and 815) and by RASs designed for the alpha 1 (RAS 806) or alpha 5 (RAS 812) species. By contrast, the alpha 5 HPV51 and the alpha 6 HPV66 were only detected with RASs corresponding to the same species (RAS 813 and RAS 811, respectively). Similarly, HPV18 that belongs to the alpha 7 species was detected with two RASs of the same species (RAS809 and 810). HPV6 that belongs to the alpha 10 species was identified by RASs of the same (RAS 810) and other (RAS 814 and 815) species. HPV42 and 53 were detected by RASs of families alpha 1 (RAS 806) and alpha 6 (RAS 811), respectively. HPV18 and 45 were tested together, and each HPV was detected by its corresponding RAS (RAS809 for HPV45 and RAS810 for HPV18) without any ambiguity (data not shown). These findings indicate that one single RAS can detect and identify several HPVs; however, the association of RASs with the homonym sequences improved the genotyping of HPVs on their own or in mixtures.

**Table 5:** Identification of HPVs by reconstructed ancestral sequences.

		Reconstructed ancestral sequences tiled on the microarray^a^									
Mixture	HPV Genotype(species)	805 (α 8)	806 (α 1)	808 (α 10)	809 (α 7)	810 (α 7)	811 (α 6)	812 (α 5)	813 (α 5)	814 (α 9)	815 (α 9)
A	16 (α9)		43.3^b^					59.9^b^		57^b^	39.9^b^
	51 (α 5)								88.9^b^		
	66 (α 6)						75.7^b^				
	108 (γ 6)										
B	6 (α 10)			54.3^b^						48.6^b^	45.7^b^
	18 (α 7)				54.1^b^	79.6^b^					
	42 (α 1)		51.7^b^								
	53 (α 6)						99.1^b^				

a The species corresponding to the RAS is given in parenthesis.

b The call rate value for each RAS is indicated when a sequence was identified.

### 4. Detection and genotyping of HPVs in clinical samples by RMAs

We assessed whether this RMA method could be used to identify HPVs in clinical samples. A set of 40 DNAs extracted from cervical smears with normal (n = 3) and abnormal (8 Atypical Squamous Cells of Unknown Significance (ASC-US), 1 Atypical squamous cells cannot exclude HSIL (ASC-H), 11 Low-grade Squamous Intraepithelial Lesion (LSIL) and 17 High-grade Squamous Intraepithelial Lesion (HSIL)) cytology were provided by the French HPV Reference Laboratory (Table 6). For each sample, the HPV status was first determined with the PapilloCheck genotyping kit. Most of the samples contained HPV16 or HPV18 on their own or with other LR or HR HPVs. The copy number per cell was between 0.001 and 71 for HPV16 and 2 and 362 for HPV18, as determined by quantitative real-time PCR (Table 6). The DNA preparations were treated with PlasmidSafe DNase and amplified with phi29 polymerase, and then subjected to genotyping by RMAs with homonymous sequences and RASs. All assays with the PapilloCheck genotyping kit and RMAs were performed only one time except with sample n°2012-312. This DNA was independently amplified and hybridized on RMAs three times. Call rate and standard deviation (SD) values for HPV16 were determined with homonymous, non- homonymous and reconstructed ancestral sequences. SD values ranged from 0.9 to 1.9% according to the kind of considered sequences and were similar to values obtained with a triplicate assay using cloned HPV16 DNA (table 7).

**Table 6:** Genotyping of HPV from cervical smears using the PapilloCheck kit and resequecing microarray.

				HPV identified^c^		
Samples	Cytology^a^	copy/cell^b^		Papillocheck	Resequencing Microarray (RMA)	
		HPV16	HPV18		Based on homonymesequences	Based on reconstructedancestral sequences
2012-1557	N			Neg	Neg	Neg
2012-1558	N			Neg	Neg	Neg
2012-1559	N			Neg	Neg	Neg
2012-1930	ASCUS			Neg	Neg	Neg
2012-1931	ASCUS			Neg	**103**	Neg
2012-620	ASCUS	1.6		16	16	16
2012-277	ASCUS	1.7		16, 42, 56	16, 42, 56	16, 42
2012-1660	ASCUS			35, 45, 53, 56	**30**, 35, 45, 53, 56, **67**, 73	35, 45, 53
2012-1649	ASCUS			6, 39, 42, 44/55, 45	6, 42, 45, 55, **101**	6, 42, 45, 55
2012-1620	ASCUS			40, 43, 44/55, 52, 56	40, 43, 52, 55, 56	43, 52, 55, 56
2012-1642	ASCUS			40, 42, 51, 53, 59, 73	35, 40, 42, 51, 53, 59, **67**, 73	40, 42, 51, 53, 59, **67**, 73
2012-249	ASC-H	0.9		16	16	16
2012-328	LSIL	0.5		16	16	16
2012-146	LSIL		361.5	18	18, **54**, **74**	18, **54**, **74**
2012-133	LSIL		11.3	18	18	18
2012-352	LSIL	4.2		16, 35	16, 35, **67**	16, 43, 67
2012-354	LSIL	84		16, 39	16, **34**, 39	16, 39
2012-601	LSIL		2.9	18, 33, 66	18, 33, 66	18, 33, 66
2012-351	LSIL	3.02		16, 33, 52, 66	16, **34**, 66	16, **34**, 66
2012-1940	LSIL			40, 42, 52, 66	**30,** 40, 42, 52, **54**, 66, 82, **91**	40, 52, 82, 66
2012-1607	LSIL			42, 51, 53, 66	42, 51, 53, 66	42, 51, 66
2012-445	LSIL	21		16, 39, 42, 59, 66	16, **32**, 39, 42, 59, 66	16, 39, 42, 59, 66
2012-1673	LSIL			33, 42, 51, 52, 66	42, 51, 52, **54**, 66	51, 52, 66
2012-451	HSIL	0.81		16	16	16
2012-495	HSIL	0.47		16	16	16
2012-622	HSIL	3.7		16	16	16
2012-234	HSIL	3.6		16	16	16
2012-312	HSIL	2.6		16	16	16
2012-499	HSIL	5.0		16	16	16
2012-248	HSIL		2.1	18	18	18
2012-314	HSIL	0.8		16, 42	16	16
2012-243	HSIL	0.05		16, 56	16, 56	16, 56
2012-266	HSIL	0.001		16, 56	16, 56	16, 56
2012-610	HSIL	70.6		16, 56	16	16
2012-613	HSIL	4.02		16, 59	16, **54**, 59	16, 59
2012-292	HSIL	3.06		16, 40, 42	16, 42	16
2012-1945	HSIL			39, 56, 66	**54**, 56, 66	39, 66
2012-453	HSIL	0.52		16, 33, 42, 59	16, 33, 59	16, 33
2012-504	HSIL		58.8	18, 39, 45, 52, 56, 59, 70	18, 39, 45, 56, 59	18, 52, 45, 56, 45

a ASCUS: Atypical Squamous Cells of Unknown Significance; ASCH: Atypical squamous cells cannot exclude HSIL; LSIL: Low-grade Squamous Intraepithelial Lesion; HSIL: High-grade Squamous Intraepithelial Lesion;

b As determined by quantitative real-time PCR.

c Neg: Negative; HPV genotypes detected by RMA and unidentifiable by the PapilloCheck kit are indicated in bold; HPV genotypes not detected by at least one of the two methods are underlined.

**Table 7:** Repeatability of RMA assay with cloned HPV16 DNA and biological sample with HPV16 monoinfection.

		HPV16			
		Cloned HPV DNA		Sample n°312	
HPV sequence tiled on RMA		Call rate^a^	Standard deviation^b^	Call rate^a^	Standard deviation^b^
Homonymous	HPV16	89.6	2.2	91.4	1.6
Non-homonymous	HPV30	29.2	0.45	26.1	0.9
	HPV33	28.0	1.4	18.7	1.6
Reconstructed Ancestral Sequence	RAS814	53	2.8	35.2	1.9

a Ratio between the number of bases determined and the sequence length.

b Microarray assays were done in triplicate.

The PapilloCheck genotyping kit can detect and identify 18 HR HPV (HPV 16, 18, 31, 33, 35, 39, 45, 51, 52, 53, 56, 58, 59, 66, 68, 70, 73, 82) and six LR HPV (HPV 6, 11, 40, 42, 43 and 54/55). The two methods PapilloCheck genotyping and RMA genotyping with homonymous sequences gave concordant results for 28/40 (70%) of the samples, and compatible results (one or two HPV missed by one or other method) for 12/40 (30%) of the samples. A value of k = 0.36 was obtained with the kappa test in interrogation of agreement between these two tests. PapilloCheck did not detect some HPVs found by RMAs (HPV 35 in sample 2012-1642; HPV73 in sample 2012-1660; HPV82 in sample 2012-1940); the RMAs did not detect some strains identified by PapilloCheck (HPV39 in sample 2012-1649, HPV33 and HPV52 in sample 2012-351, HPV33 in sample 2012-1673, HPV42 in samples 2012-314 and 2012-453, HPV56 in sample 2012-610, HPV40 in sample 2012-292, and HPV52 and 70 in sample 2012-504). RMAs allowed two closely related HPVs, HPV44 and 55, to be distinguished and genotyped, although they are indistinguishable by PapilloCheck (samples n° 2012-1620 and 2012-1649). No discordant result was observed (Table 6).

The RMA method with homonymous sequences appeared to be highly sensitive: HPV16 was detected in a sample in which there was only 1 copy per 1000 cells (sample 2012-266). Eighteen HPVs not identifiable with the PapilloCheck genotyping kit were detected by RMAs in 14 samples (indicated in boldface in Table 6). In particular, two gamma HPVs (HPV 101 and 103) were identified in samples 2012-1931 and 2012-1649; these HPVs were first detected in cervico-vaginal smears [22].

HPV genotyping following RMAs by homonymous sequences and RASs gave compatible results, although fewer HPVs were identified with RASs, particularly when the number of HPVs detected by homonymous sequences was high. However, in one case, HPV39 first identified by PapilloCheck, was detected only by RASs (sample 2012-1945) (Table 6).

## Discussion

Multiple broad-spectrum PCR methods have been developed for the detection of the alpha-HPV genus and most are based on HPV sequences in the L1 open reading frame. Here, we show that resequencing microarray (RMA) technology was feasible and effective for genotyping human papillomaviruses, and is thus a potentially valuable tool for epidemiology. We have demonstrated that the use of homonymous HPV sequences and reconstructed ancestral sequences (RASs), either separately or combined, allows precise molecular identification of different HPV genotypes. We validated the method first with cloned HPV DNA and subsequently for detection of HPVs in clinical samples. The RMA method used involved treatment of cellular DNA with Plasmid-Safe DNase and the universal Phi 29-based amplification; this methodology was highly sensitive for the genotyping of HPVs in clinical samples, with a detection limit of one copy of HPV16 per 1000 cells.

This RMA method allows the detection and characterization of HPV genotypes other than those tiled on the microarray. Indeed, the call rate values were a function of the percentage of divergence between the tiled sequence and the sequence present in the sample. We found that there was no substantial loss of detection signal when the tiled sequence diverged by up to 10–15% from the sequence of the pathogen in the sample. Moreover, the RASs were designed to minimizing the divergence between the tiled and the viral sequences potentially present in clinical samples. Indeed, the use of RASs allowed an improvement in the detection of several HPVs. Nevertheless, the nucleotide diversity of HPV sequences is substantial and not a single RAS was able to detect all the different HPVs present in cases of multiple infections.

We compared this RMA method to a commercial HPV genotyping kit. It must be stressed that clinical samples with more than one HPV were intentionally selected to compare the two methods. Some HPV types identified in clinical samples by the RMA method were not identified by the PapilloCheck test. This discrepancy is in part explained by the number of HPV genotypes, limited to 24, which can be identified by the PapilloCheck test. The HPVs detected by RMAs but not by PapilloCheck tests include both LR HPVs (HPV 32, 34, 54, 74, and 91) and HR HPVs (HPV30, 34, and 67). Also, HR HPV35, 73, and 82, found in some clinical samples by RMAs, were not detected by the PapilloCheck test. Conversely, HPV33, 39, 52, 56, and 70 identifiable in some samples by the PapilloCheck kit were not found by the RMA method. This presumably reflects different sensitivities of the two tests for some HPV genotypes.

Our study has some limitations. First, due to the substantial genetic diversity of HPVs with more than 170 HPV genotypes already identified [1], not all alpha or gamma HPVs were studied, and HPVs belonging to beta, mu and nu genera were not studied. Nevertheless we were able to identify HPV101 and 103 that belong to the gamma 6 species, and that were initially detected in cervico-vaginal smears [20]. To increase the coverage of the known HPV diversity, and to facilitate detection of novel genotypes, the number of RASs should be increased. Second, in the current approach, only a part of the L1 gene for each HPV genotype was targeted and tiled on the microarray. The L1 gene may be deleted from the virus associated with some genital cancers, and this would lead to false negative results. The E6 and E7 genes have been described to be highly expressed in high-grade cervical lesions and cancer. Therefore, new generation RMA tests could include sequences corresponding to the E6 and E7 genes, and this would allow detection of both viral DNA and transcripts.

The cost of a high-density microarray could appear as a limit because this technology required the synthesis of a physical mask. However, the final cost can decrease if the demand increases, as may be the case in the context of a clinical diagnostic use. Moreover, the use of a RMA of the smallest size only dedicated to detect and characterize HPVs will help to reduce the global cost of assay. Adding of supplementary homonyms and reconstructed ancestral sequences will allow developing countries to obtain a better tool for the screening of HPVs. Based on the sequences obtained, RMAs may be useful for molecular epidemiological studies of HPV infection, particularly in geographical areas where the distribution of circulating genotypes has not yet been investigated. It can be used to identify variants of HPV genotypes that differ from the prototype until 10–15%. RMA-based tests may thus be informative about the prevalence and distribution of diverse HPV genotypes in the post-vaccination era.

## Supporting Information

Table S1 **Content description of VirID V3.0 RMA.**
(DOCX)

Table S2 **RASs tiled on the resequencing microarray.**
(DOCX)
