# Supplementary material for: Resequencing Microarray Technology for Genotyping Human Papillomavirus in Cervical Smears
Source: PLoS One. 2014 Nov 10;9(11):e109301. doi: 10.1371/journal.pone.0109301 (PMC4226468; doi:10.1371/journal.pone.0109301)
Supplement: Table S1 — Content description of VirID V3.0 RMA. (DOCX) [file pone.0109301.s001.docx]

**Table S1. Content description of VirID V3.0 RMA**

| **Viral family** | **Viral genus** |
| --- | --- |
| Adenoviridae | Atadenovirus, Aviadenovirus, Mastadenovirus |
| Arenaviridae | Arenavirus |
| Arteriviridae | Arterivirus |
| Astroviridae | Arterivirus, Avastrovirus, Mamastrovirus |
| Birnaviridae | Avibirnavirus, Aquabirnavirus, Blosnavirus |
| Bunyaviridae | Hantavirus, Nairovirus, Orthobunyavirus, Phlebovirus |
| Caliciviridae | Lagovirus, Nebovirus, Norovirus, Sapovirus, Vesivirus |
| Circoviridae | Circovirus |
| Coronaviridae | Alpha, beta and Gamma coronavirus |
| Filoviridae | Ebolavirus, Marburgvirus |
| Flaviviridae | Flavivirus, Hepacivirus, Pestivirus |
| Hepadnaviridae | Orthohepadnavirus |
| Hepeviridae | hepevirus |
| Herpesviridae | Cytomegalovirus, Rhadinovirus, Percavirus, Lymphocryptovirus,  Roseolovirus, Simplexvirus, Mardivirus, Varicellovirus |
| Orthomyxoviridae | Influenzavirus A, B and C |
| Papillomaviridae | Alphapapillomavirus, Gammapapillomavirus |
| Paramyxoviridae | Avulavirus, Metapneumovirus, Morbillivirus, Pneumovirus,  Respirovirus, Rubulavirus |
| Paramyxoviridae | Henipavirus, Rubulavirus |
| Picobirnaviridae | Picobirnavirus |
| Picornaviridae | Aphthovirus, Avihepatovirus, Cardiovirus, Cosavirus, Enterovirus,  Erbovirus, Hepatovirus, Kobuvirus, Parechovirus, Sapelovirus,  Teschovirus, Tremovirus |
| Polyomaviridae | Polyomavirus |
| Poxviridae | Avipoxvirus, Capripoxvirus, Leporipoxvirus, Molluscipoxvirus,  Orthopoxvirus, Yatapoxvirus |
| Reoviridae | Coltivirus, Orbivirus, Rotavirus |
| Retroviridae | Betaretrovirus, Deltaretrovirus, Gammaretrovirus, Lentivirus,  Spumavirus |
| Rhabdoviridae | Ephemerovirus, Lyssavirus, Vesiculovirus |
| Roniviridae | Okavirus |
| Togaviridae | Alphavirus |
