# Supplementary material for: Resequencing Microarray Technology for Genotyping Human Papillomavirus in Cervical Smears
Source: PLoS One. 2014 Nov 10;9(11):e109301. doi: 10.1371/journal.pone.0109301 (PMC4226468; doi:10.1371/journal.pone.0109301)
Supplement: Table S2 — RASs tiled on the resequencing microarray. (DOCX) [file pone.0109301.s002.docx]

**Table S2. RASs tiled on the resequencing microarray.**

| RAS 805 | TAACAGGGCTACTATTGCAGGTAGTATTTATTTTTCTACACCCAGTGGGTCTTTGGTTACTTCTGATTCTCAATTGTTTAACAAACCATTTTGGATACAAAAGGCCCAGGGACATAATAATGGCATTTGTTTTGGCAATCAGTTGTTTGTTACAGTTGTAGATACCACTCGTAGTACAAACTTAACGTTATGTGCTGCTACAATGACAATACTAAGTTTAAGGAATATTTGCGGCATGTGGAAGAATATGATTTACAGTTTATATTTCAATTATGTATAATAACATTAAATGCAGAGGTTATGACATATATTCATACTATGGATCCTACATTATTAGAGGACTGGAATTTTGGTGTGTCCCCACCAGCCTCTGCTTCTTTGGAAGATACTTATAGGTTTTTGTCTAACAAGGCCATTGCATGTCAAAAAAATGCTCCCCC |
| --- | --- |
| RAS 806 | TAGTAGTGCTACTCTAGCTAGTAGTATTTATTTTCCTACTCCTAGTGGCTCTATGGTTACCTCTGATGCACAATTATTTAATAAACCATATTGGTTACAACAGGCCCAGGGACATAATAATGGTATTTGTTGGGGTAATCAATTGTTTGTTACTGTTGTAGATACTACCCGTAGTACTAACATGACTTTATGTGCTGCTACAATAAAAATACTAATTTTAAGGAATATTTACGACATGTGGAGGAATATGATTTACAGTTTATATTTCAATTATGTAAAATAACATTAACTGCAGAGGTTATGACATATATTCATACTATGAATCCTACTATTTTAGAGGATTGGAATTTTGGTGTAACCCCACCACCTTCTGGTACTTTAGAGGATACATATAGGTTTGTACAATCACAGGCTATTACATGTCAAAAGAATGCTCCTCC |
| RAS 808 | TAGTAGTAATACACTTGCTAATAGTATTTACTTTAATACCCCCAGTGGCTCTCTTGTGTCTTCTGAGGCACAGTTGTTTAATAAGCCTTATTGGTTACAAAAGGCCCAGGGACACAATAATGGTATTTGTTGGGGTAATCAATTGTTTGTTACTGTTGTAGATACTACACGTAGTACTAACATGACAGTGTGTGCAGCCACTATACTAGTACAGAATATAAACAATACATGCGACATGTGGAGGAATTTGATTTACAATTTATTTTTCAATTATGTAGTATTAAATTAACTGCAGAGGTTATGGCCTATATTCATACTATGAATCCTACAATTTTAGAAGACTGGAACTTTGGGCTATCGCCTCCCCCTAATGGTACTTTAGAAGACACATATAGATATGTGCAGTCTCAGGCCATTACATGTCAAAAGCCTACTCCTGA |
| RAS 809 | TATTCGTGCAACTCCTGGCAGTTGTGTATATTCTCCTTCTCCTAGTGGCTCTATGGTTACTTCTGATTCACAATTATTTAATAAGCCATATTGGTTGCACAAGGCCCAGGGACATAACAATGGTATTTGTTGGCATAATCAATTATTTGTTACTGTTGTAGATACTACTCGCAGTACTAATTTTACTTTGTGTGCTTCTACAATAATCCTACTAAATTTAAGGAATATACTAGACATGTGGAGGAATATGATTTACAGTTTATATTTCAATTGTGTAAAATTACATTAACTACAGATGTTATGTCATATATACATACTATGAATCCTACTATTTTGGAGGATTGGAATTTTGGTGTTACCCCACCACCTTCTGCTAGTTTAGTGGATACATATCGGTTTGTACAATCAGCTGCTATTACATGTCAAAAGGATGCTCCTCC |
| RAS 810 | TATGCGTGAAACTCCTGGCAGTTGTGTGTATTCTCCTTCTCCCAGTGGCTCTATTGTTACCTCTGATTCCCAGTTGTTTAATAAGCCATATTGGTTACATAAGGCCCAGGGCCATAACAATGGTATTTGTTGGCATAATCAATTATTTGTTACTGTGGTGGATACTACCCGCAGTACTAATTTAACATTATGTGCTTCTACAATGATCCTACTAAATTTAAGCAGTATAGTAGACATGTGGAGGAATATGATTTACAGTTTATTTTTCAGTTGTGTACTATTACTTTAACTGCAGATGTTATGTCATATATCCATAGTATGAATAGTAGTATTTTGGAAGATTGGAATTTTGGTGTTCCCCCACCACCTACTACTAGTTTAGTGGATACATATCGTTTTGTACAATCTGTTGCTATTACCTGTCAAAAGGATACTACACC |
| RAS 811 | TGGCAGGGACCCTCCTCCTAGTTCTGTATATGTTGCTACTCCTAGTGGGTCTATGATTACCTCTGAGGCTCAATTATTTAATAAACCTTATTGGTTGCAACGTGCCCAGGGTCATAATAATGGCATTTGTTGGGGTAATCAGTTATTTGTTACTGTTGTGGATACTACCAGAAGTACTAACATGACTATTAGTGCAGCTACAATAATGCAAGTAAAATTAATCAGTATCTTAGACATGTGGAGGAATATGAATTACAGTTTGTGTTTCAACTATGTAAAATTACCTTGTCTGCAGAGGTTATGGCATATTTACATACTATGAATTCTACTTTACTGGAGGACTGGAATATTGGATTGTCCCCACCAGTTGCCACTAGCTTAGAGGATAAATATAGATATGTTAAAAGCACAGCTATAACATGTCAAAAGGATCAGCCCCC |
| RAS 812 | TGGCCGTGACCCTCCTACTAGTTCTATATATTCTGCTACTCCTAGTGGCTCTATGGTTACCTCTGATGCTCAATTATTTAATAAGCCTTATTGGTTGCAACGTGCCCAGGGTCATAATAATGGCATTTGTTGGGGCAATCAATTGTTTGTTACTTGTGTAGATACTACCCGCAGTACCAACTTTACTATTAGTACTGCTACTTTAATCCAACTAATTTTAAGCAATATATTAGGCATGGGGAGGAATATGAATTACAGTTTATATTTCAATTGTGTAAAATTACTTTAACTACAGAGGTTATGGCTTATATACATACAATGAATTCTACTATTTTGGAAGATTGGAATTTTGGATTAACCTTACCTCCTTCTGCTAGTTTGGAGGATGCATATAGGTTTGTTAAAAATGCAGCTACTACATGTCAAAAGGATGCCCCTCC |
| RAS 813 | TGGCCGTGACCCTATAGATAGTTATATATATTCTGCTACTCCCAGTGGGTCTATGATAACCTCTGATTCTCAAATTTTTAATAAGCCTTATTGGTTGCACCGTGCCCAGGGTCACAATAATGGCATTTGCTGGAACAATCAGCTTTTTATTACTTGTGTTGATACTACCAGAAGTACCAATTTAACTATTAGCACTGCCACTTTACTCCAACTAACTTTAAGCAATATATTAGGCATGGGGAAGAATATGAATTGCAATTTATATTTCAATTATGTAAAATTACTTTAACTACAGAGGTTATGGCTTATTTACACACAATGGATCCTACCATTTTAGAACAGTGGAATTTTGGATTAACATTACCTCCCTCTGCTAGTTTGGAGGATGCATATAGGTTTGTTAAAAATGCAGCTACTACCTGTCAAAAGGACACCCCTCC |
| RAS 814 | TACTACTGCTACTTTAGCTAGTAGTACTTATTTTCCTACTCCTAGTGGCTCTATGGTTACCTCTGATGCACAAATATTTAATAAACCATATTGGTTGCAACGTGCACAGGGTCATAATAATGGTATTTGTTGGGGTAATCAATTATTTGTTACTGTTGTAGATACTACCCGTAGTACTAATATGTCTTTATGTGCTGCTATAATAAAAATAATAATTTTAAGGAATATTTAAGACATGGTGAGGAATATGATTTACAGTTTATTTTTCAGTTATGCAAAATAACATTAACTGCAGACGTTATGACATATATTCATACTATGAATCCTACTATTTTGGAGGATTGGAATTTTGGTTTAACACCACCTCCTTCTGGTACTTTAGAGGATACATATAGGTTTGTAACATCACAGGCTATTACTTGTCAAAAAACTGCTCCTCC |
| RAS 815 | TACTACTGCCGCTATGCAAAGTAGTGCTTTTTTTCCTACTCCTAGTGGCTCTATGGTTACCTCAGAATCTCAATTATTTAATAAACCATATTGGTTACAACGTGCACAAGGTCATAATAATGGTATTTGTTGGGGCAATCAATTATTTGTTACTGTGGTAGATACCACTCGTAGTACTAATATGACTTTATGTACTGAAGTAATAAAAATGAAAATTTTAAGGAATATCTAAGACATGTTGAAGAATATGATTTACAGTTTATTTTTCAGCTATGCAAAATAACACTAACTGCAGATGTTATGACATATATTCATACTATGAATCCAGATATTTTAGAGGATTGGCAATTTGGTTTAACACCACCTCCTTCTGGTAGTTTACAGGACACATATAGGTTTGTTACCTCTCAGGCTATTACTTGTCAAAAAACAGCACCTCC |
